# Non-technological barriers: the last frontier towards AI-powered intelligent optical networks

**DOI:** 10.1038/s41467-024-50307-y

**Published:** 2024-07-17

**Authors:** Faisal Nadeem Khan

**Affiliations:** 1 Tsinghua-Berkeley Shenzhen Institute, Tsinghua University, Shenzhen, China; 2 Institute of Data and Information, Tsinghua Shenzhen International Graduate School, Tsinghua University, Shenzhen, China

**Keywords:** Electrical and electronic engineering, Fibre optics and optical communications

## Abstract

Machine learning (ML) has been remarkably successful in transforming numerous scientific and technological fields in recent years including computer vision, natural language processing, speech recognition, bioinformatics, etc. Naturally, it has long been considered as a promising mechanism to fundamentally revolutionize the existing archaic optical networks into next-generation smart and autonomous entities. However, despite its promise and extensive research conducted over the last decade, the ML paradigm has so far not been triumphant in achieving widespread adoption in commercial optical networks. In our perspective, this is primarily due to non-addressal of a number of critical non-technological issues surrounding ML-based solutions’ development and use in real-world optical networks. The vision of intelligent and autonomous fiber-optic networks, powered by ML, will always remain a distant dream until these so far neglected factors are openly confronted by all relevant stakeholders and categorically resolved.

## Abstract (web summary)

The author sheds light on critical non-technological barriers that significantly limit the broad utilization of machine learning in optical networks and presents several prospective solutions. Various pathways are discussed for the evolving machine learning potential for its desired penetration, credibility, and impact in real-world optical networks.

## Introduction

Data-driven ML models have arisen as potent tools for addressing numerous intricate challenges in optical communications and are being envisaged as an enabler for future efficient, intelligent and reliable network infrastructures^1,2^. In the last few years, industry as well as academia has witnessed a significant increase in research endeavors to harness and capitalize on ML across different facets of fiber-optic communications ranging from designing of network components^3–7^ to compensating critical transmission impairments^8–10^ to predicting data traffic flow patterns in networks^11,12^. However, despite unprecedented interest in this field over the past decade, the developed ML methods have unfortunately not yet attained anticipated deployment, credibility and impact in real-world fiber-optic networks, barring the successful implementation of some non-network related business use-cases (e.g., customers’ churn prediction, customers’ segmentation, etc.) by the operators.

**Fig. 1:**
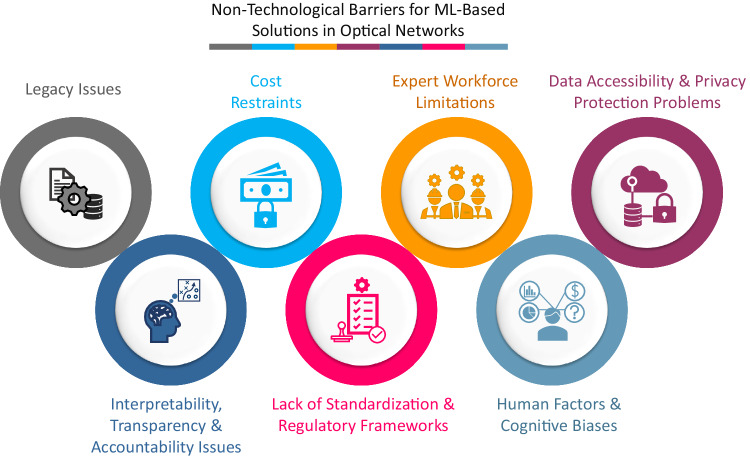
Critical non-technological limiting factors. Seven key non-technological challenges faced by ML-aided methods in fiber-optic networks.

In our viewpoint, the key impediment towards widespread application of ML-aided methods in commercial fiber-optic networks is the existence of several pending non-technological limiting factors that are crucial from practical networks’ perspective but have been largely ignored by the relevant stakeholders. In this article, we systematically identify seven non-technological barriers, as shown in Fig. 1, which are currently hindering broad deployment of ML-based solutions in real-world optical networks. These include: (1) Prevalence of legacy systems and processes, (2) Cost restraints, (3) Expert workforce limitations, (4) Data accessibility and privacy protection problems, (5) Explainability, transparency and accountability issues of ML models, (6) Lack of standards and regulatory policies for ML-aided methods, and (7) Human factors and cognitive biases.

To substantiate our viewpoint, we take five major ML application areas in optical networks, namely (i) Network failures management, (ii) End-to-end (E2E) communication system optimization, (iii) Lightpaths’ quality of transmission (QoT) estimation, (iv) Optical performance monitoring (OPM), and (v) Network security management, as examples and qualitatively and quantitatively highlight how the afore-mentioned seven non-technological challenges greatly diminish the scope of employing developed ML-aided methods in practical fiber-optic networks. More profoundly, we provide an extensive set of solutions which can be instrumental in overcoming all the non-technological issues raised above, thus paving the path for broad adoption of ML-enabled efficient processes in future commercial optical networks. Due to timely nature of the addressed topic and an up-to-date coverage of several pertinent problems, this article will likely be interesting for a wide range of audience and can help stimulate further research by the community.

## Major non-technological challenges for ML-based solutions

In this section, we discuss each individual non-technological barrier and elucidate our viewpoint with the help of example use-cases from real fiber-optic networks.

### Legacy issues

Conventional non-ML solutions in optical networks offer a well-established ecosystem due to decades of successful deployment and operation history^13^. Notably, significant investments were dedicated to their development over time. This lessens the motivation to switch to alternative ML-based tools, after all, why fix something that is not broken? A 2021 study report by TM Forum (which is a global alliance of 850+ telecommunication companies including network operators, network equipment vendors, network management consultancies, etc.) that draws on a survey of 42 global operators showed that the issue of prevalence of legacy solutions was ranked as the topmost barrier to ML-aided autonomous networking by the survey respondents^14^.

While pitched against the traditional legacy tools, ML-assisted methods also suffer from reputation and trust challenges since it is sometimes argued by the optical network operators that the ML-based solutions developed in research may have little relevance and usefulness in practical fiber-optic networks.

#### Example network use-case: lightpaths’ QoT estimation

A prime example of legacy issues is the ML-aided QoT estimation^15–17^ operation in optical networks, which has traditionally been dominated by non-ML approaches such as Gaussian noise (GN) model^18^ and its variants^19^. ML-based QoT estimation methods, despite offering significant advantage in scenarios involving certain uncertainties about link parameters values^20,21^,  have not been successful yet in achieving broad adoption in current fiber-optic networks and it may take a while before these techniques are deemed suitable substitutes for their legacy counterparts.

### Cost restraints

As ML models essentially rely on data, having sufficient representative network data is a prerequisite for guaranteeing good model performance. However, the data generation and analysis processes demand substantial investments, e.g., for installing monitoring equipment (like optical channel monitors (OCMs), optical time-domain reflectometers (OTDRs), optical spectrum analyzers (OSAs), etc.) ubiquitously across the network; for securely storing large amount of network data; for procuring fast computational resources to process big data using ML algorithms; for acquiring an extended set of software tools; for employing additional skilled workforce, etc.^22^. What makes the matters worse is the unclear business impact of such sizable investments since the network operators often find it hard to translate ML models’ statistical metrics (e.g., accuracy) into relevant business value. The cost issues mentioned above pose major hindrance to ML-aided methods’ deployment in optical networks. Based on a 2022 worldwide survey of 78 network operators, which was jointly conducted by Light Reading Inc. (a New York-based telecommunications industry information company) and 4 key optical transport network suppliers, i.e., Ciena, Fujitsu, Infinera, and Juniper networks, while the survey respondents envisioned several benefits of ML-powered tools for open, automated and programmable transport networks, 36% of them identified high costs as the main obstacle towards their adoption^23^.

#### Example network use-case: optical performance monitoring

Consider the OPM process in fiber-optic networks that is conventionally realized through low-cost ubiquitously-deployed OCMs, which typically comprise of a simple tunable band-pass filter or a diffraction grating followed by a single photodetector to monitor a few channel parameters like optical power, wavelength, and out-of-band optical signal-to-noise ratio (OSNR)^24^. Although ML-based alternative OPM techniques^25,26^ have successfully demonstrated multi-parameters monitoring for several wavelength-division multiplexed (WDM) channels, their vast deployment in optical networks still remains limited. The reason for this is that such data-driven OPM solutions necessitate new mechanisms for assembling a variety of training data as well as extra data analysis tools, all of which incur additional costs, thus restraining the network operators to switch to this new paradigm despite its obvious technical supremacy.

### Expert workforce limitations

ML-assisted operation and management of optical networks is still at its infancy. That is why, there is currently a shortage of well-trained professionals proficient in both ML techniques and domain expertise related to optical communications and networking. This dearth of expert workforce has been reported by several industry stakeholders, such as Huawei Technologies^27^ and Colt Technology Services^28^, where the former proposed an operating model to retrain and transform the skills of a certain fraction of original workforce in a telecommunication company (e.g., converting the operations personnel into data analytics engineers) to cope with the ML workforce limitations.

Another crucial aspect, as the optical network operations aim to transition towards new “human + machine” collaboration model, is that the existing workforce is accustomed to traditional non-ML tools and thus it will be quite hard for it to work and cooperate with network devices/equipment controlled by artificial intelligence (AI). This is problematic because in many critical optical network operations, necessitating very high level of reliability, the workforce is required to seamlessly interact with ML models and fully trust their recommendations and decisions. The lack of user-friendly ML tools that could help the unaccustomed workforce easily apply ML methods further aggravates the situation. The above-mentioned workforce challenges ensure that a transition towards ML-aided or hybrid solutions in fiber-optic networks is going to be a lengthy process.

#### Example network use-case: end-to-end communication system optimization

Consider the fiber-optic communication system optimization process, where conventionally the individual system components like transmitter, receiver and transmission link are separately designed and optimized in a modular fashion by different teams of engineers, often leading to suboptimal E2E system performance^29^. In contrast, an ML-assisted E2E learning and optimization approach optimizes various transceiver blocks, such as coding, pulse shaping, modulation, equalization, demodulation, decoding, etc., jointly such that the errors between transmitted and final received bits are minimized. Over the last few years, several E2E solutions^29–31^ involving artificial neural networks, autoencoders, etc., have been experimentally demonstrated and despite offering clear performance advantages, their application in commercial optical networks remains nonexistent due to current scarcity of skilled professionals with multifaceted expertise in ML, digital signal processing (DSP), and optical communications, all of which are imperative for realizing ML-based E2E optimization of fiber-optic communication systems.

### Data accessibility and privacy protection problems

For developing effective ML-aided solutions, it is necessary to access characteristic data sets from actual optical networks. However, realistically, it is hard to achieve that because of several practical reasons. Firstly, the mechanisms for seamless global access to relevant sources of data are not fully established yet, e.g., due to data ownership constraints, data sets size, bandwidth limitations to transport large volumes of data, etc. Secondly, the terms-of-use (ToU) for such shared data are not clearly defined yet. This has resulted in various stakeholders isolating their data and setting boundaries for data sharing in order to protect their own commercial interests. Thirdly, the rules and regulations for protecting the privacy and anonymity of shared data have not yet been enacted in current optical networks environment.

Due to above-mentioned constraints, there are presently only handful examples of real-world optical networks data sets that are openly accessible to solution developers. These include Microsoft wide-area optical backbone network performance monitoring data^32^ released in 2017, Alibaba production optical transport network QoT data^33^ released in 2023, Germany50 and pan-European GÉANT optical backbone networks traffic data^34^, etc. However, these data sets correspond to certain specific use-cases only (i.e.,  OPM, QoT estimation, and network traffic flow prediction, respectively) and are also limited in scale.

#### Example network use-case: physical layer security management

Consider the ML-assisted physical layer security management operation^35–37^ in fiber-optic networks that is not realizable through localized actions only and necessitates network-wide sharing of data related to security incidents between entities belonging to various network domains. Unfortunately, effective mechanisms to access and use the required sensitive data as well as the protocols to assure data privacy in such collaborative network applications are still missing.

### Interpretability, transparency and accountability issues

The lack of comprehensible explanation of the decisions made by the ML algorithms (which typically employ a “black-box” methodology) is a big hurdle in adopting ML-based solutions for mission-critical operations in commercial fiber-optic networks because it is not preferable in practice to employ a solution without really understanding how and why it works. Optical network operators are particularly interested in knowing how different factors affect the prediction results and may sometimes opt for simpler and intuitive non-ML models with inferior performance in exchange for having better insights. Recently, we have seen network operators and solution developers worldwide beginning to take significant interest in incorporating explainability in their ML-aided operations and decision-making. For example, Nokia Bell Labs^38^ proposed a ML-enabled proactive fiber breaks detection mechanism in optical networks that additionally provides interpretable decision-making rules. Similarly, in a 2021 policy paper^39^, Deutsche Telekom provided guidelines to its solution developers on how to increase the degree of comprehensibility of their ML-based solutions.

On the other hand, the absence of transparency in ML-assisted tools makes it hard to scrutinize and detect any potential discrepancies. Moreover, it leads to accountability problems, e.g., in the event of a false decision made by a ML-based solution, it is often impossible to determine whether or not the issue lies with ML model, the training data sets employed, the entities which obtained data, or the equipment used.

#### Example network use-case: network failures management

Consider the process of detecting faults in optical networks, where the conventional tools establish certain specific threshold levels and trigger some alarms whenever the set levels are surpassed, thus enabling a primitive but intuitive fault detection mechanism^2^. On the other hand, by leveraging large amount of components data, links data, and network operational data, the ML-aided fault management approaches have successfully demonstrated several advanced features including proactive fault detection, fault classification, fault localization, fault root cause analysis, and preventive maintenance^2,40–42^. However, such ML-based fault management solutions have not yet received broad acceptance from optical network operators because they hardly provide any insights about how certain decisions were reached and why exactly they shall be trusted.

### Lack of standardization and regulatory frameworks

As ML techniques for fiber-optic networks are still evolving, there is currently a lack of consensus among the stakeholders on a range of issues related to the standardization of: data generation processes, data sets specifications for various network use-cases, data structures and formats, ML models’ performance metrics, ML models’ performance evaluation procedures, etc. Another issue closely related to standardization is the use of highly-specialized but little-standardized terminology for ML-based solutions, which is problematic for novices, e.g., technicians in the field.

It is worth mentioning here that in case of wireless networks, several global standardization organizations have taken serious steps in the past few years to address ML-related standardization challenges. For example, in 2017, International Telecommunication Union Telecommunication Standardization Sector (ITU-T) initiated a Focus Group on ML for Future Networks including 5G (FG-ML5G) that aimed to identify the standardization gaps of ML for 5G and beyond networks^43^. Similarly, in 2017, China Telecom together with Huawei Technologies and other partners set up a work group named Experiential Networked Intelligence (ENI) in European Telecommunications Standards Institute (ETSI) to facilitate the formulation of standards for ML applications in wireless networks^44^. Unfortunately, for optical transport networks, the efforts for standardizing the data generation and processing processes for different ML applications are still at a nascent stage. To this end, in 2019, National Institute of Standards and Technology (NIST) hosted a workshop^13^ which highlighted the emerging need of standardizing the relevant data sets for ML-aided fiber-optic networks.

Apart from standardization, there is also a dearth of regulatory policies pertaining to the regulation of “data market” as well as implementation, fair assessment, and anti-discrimination validation of ML models. Even worse, to the best of author’s knowledge, there aren’t any regulatory bodies in place yet to provide expertise and oversight on forthcoming legal challenges to ML-enabled solutions in optical networks.

#### Example network use-case: lightpaths’ QoT estimation

A relevant example of lack of standardization and regulatory frameworks is the ML-aided lightpaths’ QoT estimation, where the ML algorithms are trained to learn the complex mapping between the feature vectors, comprising of few selected parameters of the link/signal, and the lightpath’s chosen QoT metric^15–17^. However, there is presently no standardization of the used feature vectors and various proposed solutions apply dissimilar parameter sets, leading to divergent QoT estimation performances. Similarly, there is no standardization of the QoT metric itself and several alternatives like lightpath’s feasibility class (i.e., a binary variable), OSNR, electrical signal-to-noise ratio (ESNR), *Q*-factor, bit-error ratio (BER), error vector magnitude (EVM), etc., have been considered^45^. Furthermore, there are currently no bodies existing to regulate the used data sets and the ensuing data-driven models for predicting QoT. Due to above shortcomings, the optical network operators have no real means available to fairly compare different ML-based QoT estimation methods, which in turn reduces their adoption prospects.

### Human factors and cognitive biases

Unlike conventional analytical approaches used in fiber-optic networks with certain fixed performance, the results of ML-based solutions are strongly dependent on which data points are included and which are ignored (on purpose or by accident) by the human developers, which is problematic since it may disallow objective and human-independent performance measure. Moreover, since ML algorithms are essentially trained by the humans, who naturally gain knowledge, technical skills, experience and intuition around certain processes, equipment and tools, it may result in some cognitive biases (a term in psychology that describes the tendency of people’s experiences and feelings to influence their judgment^46^) and hence lead to distorted predictions.

The presence of human factors and intentional/unintentional biases makes it hard for optical network operators to completely trust ML-based prediction results especially while taking critical decisions. In a 2023 study report^47^ published by the Body of European Regulators for Electronic Communications (BEREC), the results of a survey of 7 European network operators, such as Telefónica Germany, Koninklijke PTT Nederland, Telefónica, S.A., etc., showed that the respondents ranked undetected data biases as the topmost area of concern since they could entail misleading results.

#### Example network use-case: network failures management

A prime example of human factors and cognitive biases is the ML-based fault detection operation^40–42^ in fiber-optic networks, where it is not always possible to completely automate the data generation process. For example, to assign a “normal” or “faulty” label to a given acquired data sample from a certain network device, the involvement of a subject matter human expert who could give a higher-level interpretation is inevitable. In such cases, there is always a risk that the annotation of data used in the learning process and consequently the performance of ML model may become vulnerable to humans’ inferences. Similarly, the performance of ML-aided fault detection tools may vary depending upon the nature of data employed (e.g., time/frequency/polarization domain data, optical/electrical domain data, etc.) as well as the type of ML algorithm applied, all of which are strictly humans’ prerogative and are often dictated by their prior experience, degree of familiarity, convenience, etc. The dependence of ML-based fault management solutions’ performance on humans’ traits makes them a less credible choice for optical network operators.

## Prospective solutions for non-technological limiting factors

Below, we propose some potential strategies that can aid in resolving the pending non-technological challenges discussed above, thus clearing the path for vast utilization of ML-aided methods in upcoming commercial optical networks.

### Addressing legacy issues

The cost-benefit comparisons of ML-based and legacy solutions can be used to entice the network operators that persisting with the legacy tools will in fact turn out to be more expensive in the long run since employing modern ML-aided methods can greatly enhance the network performance and hence offer better value. According to a 2022 analysis report by McKinsey & Company^48^, the field operations of most telecommunication companies account for 60−70% of their operating budgets. In view of this, the optical network operators can be convinced that the automation of network processes through ML-powered intelligent solutions can help reduce humans’ involvement, streamline network operations, and enable self-healing networks that are more resilient and reliable, thus bringing real and rapid cost benefits.

Furthermore, the network operators can be persuaded that in today’s world of breakneck innovation race, it may be too risky for them to rely on outdated legacy tools since it can lead to competitive disadvantage and hamper their future growth. Proven examples of successful adoption of ML in network business use-cases, e.g., ML-assisted customers’ behavior analysis, can particularly be evinced in this regard to affirm what ML is realistically able to accomplish.

### Relaxing cost restraints

The cost restraints can possibly be reduced through following useful measures. Firstly, instead of collecting network data through ubiquitously-deployed stand-alone monitoring devices, reliance can be made on rich monitoring data that is readily available in a digital coherent receiver^49,50^ (e.g., received optical power levels, OSNR, accumulated chromatic dispersion, polarization-mode dispersion, fiber nonlinear coefficient, etc.) as a by-product of standard DSP modules. Secondly, simulation data (generated using certain software tools, such as VPIphotonics^51^) can be considered as an auxiliary data source to decrease the dependence on expensive optical networks data. Although such synthetic data may not be an exact replacement of actual data from networks, it can be quite valuable during pre-training process of ML algorithms that can subsequently be improved utilizing some minimal real networks data. Finally, priority can be made in using those specific ML models that can learn and predict accurately even when limited training data is available, e.g., transfer learning^52^.

### Tackling expert workforce limitations

To address expert workforce limitations, both industry and academia will need to make serious efforts and investments in developing interdisciplinary education programs that can help prepare workforce having fundamental knowledge of communications engineering, photonics, and ML theory along with necessary programming and algorithms training expertise. A practical example of such an initiative is the European Union-funded MENTOR project^53^ that aims to train early-stage researchers to foster their skills in the interdisciplinary field of ML-aided multi-band optical communications. The project involves a consortium of four industrial partners (i.e., Coriant Germany and Portugal, Orange Labs, and Telecom Italia Mobile) and three European academic institutes (i.e., Technical University of Denmark, Sant’Anna School of Advanced Studies, and Polytechnic University of Catalonia) and intends to produce a new generation of optical communications researchers that is well-equipped with necessary ML knowledge.

Apart from researchers, the industry might also consider raising and maintaining sufficient skilled workforce of engineers and technicians, where besides domain-specific knowledge, these professionals will be expected to have expertise in constructing and troubleshooting testbeds to generate relevant data sets for the training of ML models. Such collaborative initiatives aimed at developing ML skills can optimize resource utilization, reduce financial strain on individual partners, and expand the available technical expertise pool.

### Solving data accessibility and privacy protection problems

For enabling worldwide access to pertinent optical networks data, following steps can be taken. Firstly, since the real networks data is considered a valuable entity by the network operators, clear benefits/rewards of sharing such data must be identified in order to incentivize data sharing. Secondly, industry stakeholders such as network operators, network equipment vendors, solution developers, etc., shall come together to establish protocols and define clear ToU for the proprietary network data to encourage trustworthy data sharing.

On the other hand, for preserving data privacy, ML frameworks such as federated learning^54^ shall be explored, which can allow a cohort of network operators to collaboratively build a common ML model without actually sharing any sensitive data. In this case, each network operator can first train its own ML model utilizing its network data and then send the model’s parameters (instead of raw sensitive data) to a central server which may aggregate the models sent by all the network operators to build a common shared model and finally send its parameters to the individual network operators. This can help allay data privacy concerns, ensuing in more enhanced partnerships between various stakeholders.

### Overcoming interpretability and accountability issues

To address interpretability flaw of ML-aided methods for optical networks, one useful approach can be to employ comparatively simple ML models for applications that are not too complicated. Such reduced-complexity “glass-box” ML models will inherently be more transparent to inspection and may include examples like decision trees, Bayesian networks, sparse linear models, etc.^55^. Conversely, many sophisticated network applications warrant using more intricate and intrinsically non-transparent deep learning (DL) algorithms and in those scenarios interpretability techniques^56^, such as local interpretable model-agnostic explanations (LIME), layer-wise relevance propagation (LRP), deep learning important features (DeepLIFT), etc., can be applied to potentially elucidate the behavior of developed DL models.

Similarly, to deal with the accountability issues of ML-aided tools for optical networks, some possible solutions include: (i) Achieving accountability through interpretability methods, where the interpretability can act as a helper to accountability to better understand why a particular ML model made a certain false prediction, (ii) Ensuring complete transparency of the development process by disclosing how the ML model came to be including the details of the data sets used, ML model’s choice, training procedure, ML model’s parameters after training, etc., thus removing all opacity barriers and helping understand why a certain unexpected behavior actually occurred, and (iii) Employing post-hoc inspection methods^57^ that accept an already-developed ML model to be a black-box and apply certain sets of inputs to it and by observing the model’s input-output relationship try to identify its properties and determine whether it has behaved correctly or not.

### Realizing standardization and regulatory frameworks

To meet emerging standardization challenges of ML-aided methods for optical networks, there shall be joint efforts by industry and global standardization organizations, such as Institute of Electrical and Electronics Engineers Standards Association (IEEE-SA), NIST, International Organization for Standardization (ISO), etc., to define standards for data generation processes, data sets specifications for various network applications, performance metrics of ML models, etc. The testing conditions for ML models must also be standardized rather than leaving them on developers’ discretion, who may select conditions that present a model in a favorable light.

Similarly, the industry and governmental bodies shall jointly formulate regulatory policies for data market and ML models. This may include attempts such as obligating the “datasheets” for all generated data sets documenting the reasons behind producing a given data set and its generation method, its information content, potential data biases, recommended tasks for its application, and any legal considerations associated with its use. Likewise, for a developed ML model, a “report card” (similar to supplier’s declaration of conformity (SDoC) document^58^ used in some other fields like electronics products and telecommunications equipment) can be warranted disclosing its performance in a variety of operating conditions, detailing its performance evaluation procedures, providing the context in which the model is suggested to be used, etc. The proposed datasheets and report cards can possibly be certified by some independent bodies to enhance trust among different stakeholders.

The realization of above-mentioned standardization and regulatory frameworks will go a long way in ensuring objective and fair assessment of the developed ML-based solutions as well as increasing their operability across various optical networks.

### Neutralizing human factors and cognitive biases

To mitigate human factors and unwanted biases and strengthen trust in ML-aided methods for fiber-optic networks, following measures can be helpful. Firstly, the solution developers may consider forming a diverse team with wider variation of experiences for data generation processes as well as may aggregate inputs from multiple data sources to minimize the risk of data biasness. Secondly, the developers shall promote complete transparency by providing datasheets that clearly specify whether the data sets on which the developed ML models are trained and tested were rigorously checked for any prejudices and biases and what attempts were made to ensure that they were indeed fair and representative. They may also provide links to the original data sets so that the developed tools could be independently verified for fairness. Thirdly, the hidden biases in the data sets can be spotted using certain statistical tests and subsequently eliminated by applying a combination of pre-processing (e.g., randomized mapping to transform a raw biased data set into a new unbiased one^59^), in-processing (e.g., adding a bias-aware regularization term in the loss function^60^), and post-processing (e.g., equalized odds post-processing^61^ which punishes ML models that perform well only for a particular data class) debiasing techniques.

Table 1 shows which specific ML-enabled applications’ deployment prospects in fiber-optic networks are adversely affected by each individual non-technological issue discussed above. It also provides a brief summary of the proposed solutions for each of these limiting factors. Among different affected applications mentioned in the table, ML-assisted photonic components design is unique in the sense that it is a device-level application in contrast with other link/network-level ones. In the past few years, ML techniques have been actively considered for the design and performance optimization of various optical network devices, such as Erbium-doped fiber amplifiers’ (EDFAs) gain modeling^62^, Raman amplifiers’ gain profile design through optimization of pump powers and wavelengths^63^, directly-modulated lasers’ modeling^64^, lasers and frequency combs’ phase and frequency noise characterization^65^, etc., and have shown great potential. However, compared with conventional physics-based device modeling and design approach, broad adoption of data-driven ML methods is still constrained due to the presence of a number of technical challenges (e.g., the burden of generating gigantic amount of labeled data through simulations and physical experiments to train ML models^5^, risk of unexpected device behavior since ML models even if trained using carefully-assembled data sets may contain singular points at which their predictions can diverge^66^, inflexibility of the ML-based approach since even minor device modifications may require generation of entire training data from the scratch^66^, etc.) as well as various persisting non-technological barriers including explainability and accountability issues^67^, expert workforce limitations, absence of essential standardization frameworks^68^, etc., as mentioned in Table 1.

**Table 1:** Sample ML-aided applications that are limited by the individual non-technological challenges and a summary of the potential solutions to those challenges

Type of non-technological challenge	Example ML-aided applications affected	Summary of proposed solutions
Legacy issues	• Quality of transmission estimation^15–17,45^ • Network failures management^40–42^ • Adaptive allocation of network resources^73–75^ • End-to-end communication system optimization^29–31^	• Conveying long-term cost-saving potentials of ML-enabled intelligent solutions to the network operators • Establishing communication channels between solution developers and network operators to convey what ML-aided tools are able to achieve realistically and how they can help gain advantage over other competitors
Cost restraints	• Optical performance monitoring^25,26^ • Quality of transmission estimation • Network security management^35–37^	• Curtailing data costs by exploiting data readily available in coherent receivers as a by-product of DSP modules • Exploiting cheap synthetic data from simulation tools • Reducing data dependency by applying less data-hungry ML algorithms
Expert workforce limitations	• End-to-end communication system optimization • Network failures management • Photonic components design^3–7,62–65^	• Developing interdisciplinary education programs supported by industry and academia to prepare expert workforce adept in both optical communications and ML • Introducing collaborative ML skills development programs backed by industry stakeholders to produce required workforce of field engineers and technicians
Data accessibility and privacy protection problems	• Network security management • Network failures management	• Identifying clear benefits of sharing real networks data to incentivize the network operators to share their data • Defining protocols and clear ToU for proprietary network data to encourage trustworthy data sharing • Protecting data privacy in collaborative network applications through federated learning framework
Interpretability, transparency and accountability issues	• Photonic components design • Fiber nonlinearity compensation^8–10^ • Network failures management	• Using comparatively simple but comprehensible ML methods for less complicated network applications • Applying interpretability frameworks to explain the behavior of intricate and non-transparent DL methods developed • Enabling accountability by disclosing all information related to data generation processes, details of the data sets used, and ML models’ development procedures
Lack of standardization and regulatory frameworks	• Quality of transmission estimation • Short-reach data centre networking^76,77^ • Photonic components design	• Defining standards for data generation processes, data sets specifications for various applications, ML models’ performance metrics and testing conditions, etc., through cooperation of industry and standardization organizations • Formulating regulatory policies for data market (e.g., obligating datasheets for all data sets) and ML models (e.g., warranting report cards for developed ML models) through joint efforts of industry and governmental bodies
Human factors and cognitive biases	• Network failures management • End-to-end communication system optimization (e.g., which specific transmission factors are not modeled in the end-to-end optimization process is decided by the humans leading to certain performance penalties)	• Diversifying workforce responsible for data generation processes and aggregating inputs from multiple data sources to minimize the risk of data biasness • Providing datasheets that confirm checking and absence of any data biases and also give links of original data sets so that developed tools could be independently verified • Detecting hidden biases in data using statistical tests and eliminating them through various debiasing techniques

## Degree of difficulty of non-technological challenges

**Fig. 2:**
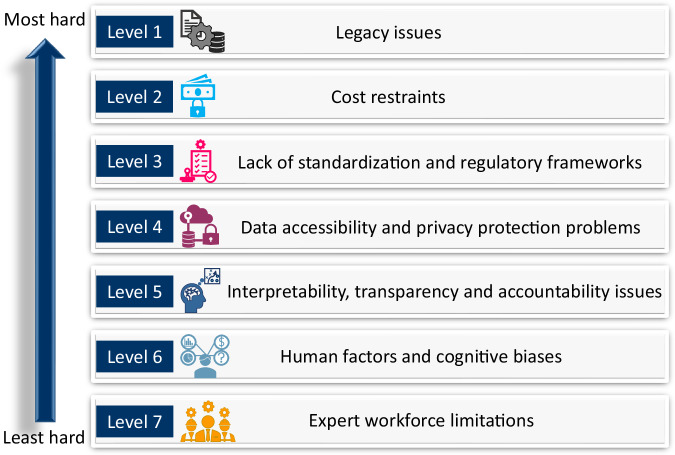
Ranking order of considered non-technological barriers. Difficulty levels of seven key non-technological challenges hindering vast utilization of ML-aided methods in optical networks.

In Fig. 2, we rank seven considered non-technological challenges based on the degree of difficulty faced in their resolution. We draw these conclusions based on our own perspectives as well as on some insights from the relevant industry^13,14,23,47^. In our viewpoint, the two biggest challenges to overcome, in order, are the legacy issues and cost restraints due to associated financial implications of overhauling the existing optical networks infrastructure, while given the fact that conventional network operators typically have a sluggish revenue growth^69^ which greatly shrinks their prospects of shifting to new ML paradigm.

The realization of standardization and regulatory frameworks, that is indispensable for developing universally-operable ML-aided methods for optical networks, is the next big problem to solve since such frameworks warrant joint initiatives by industry, standardization organizations and regulatory bodies, which have so far been lacking unfortunately^13^. The fourth critical challenge, in order, is providing global access to relevant sources of data while preserving data privacy and anonymity. However, establishing such data sharing mechanisms and defining clear data-usage terms for proprietary network data are still a work in progress.

The next two problems, in order, are the interpretability/accountability issues of ML models and the presence of intentional/unintentional biases, which although do not fundamentally deter the implementation of ML-aided tools, their solution is vital for actualizing credible decision-making processes in optical networks. Recent progresses in explainable AI (XAI) research^56,70^ and data debiasing techniques^71,72^ will particularly be helpful in addressing these two challenges, respectively.

Lastly, the expert workforce limitation is expected to be a pressing problem mainly in the short-term as the recent interests shown by the industry, academia and governments around the world to foster basic ML knowledge and skills will likely be consequential in dealing with the trained workforce scarcity in due course.

## Summary and outlook

ML is envisioned to be the key driving force for radical innovations in upcoming optical networks. However, vast utilization of ML-aided methods in real-world optical networks presently remains limited due to a number of so far ignored non-technological factors. In this article, we highlighted and ranked seven such issues and with the aid of several relevant network use-cases demonstrated that how these factors greatly reduce the deployment scope of ML-aided tools in practical networks. We also proposed several strategies to overcome these non-technological barriers, thus facilitating widespread adoption of ML-powered solutions for cognitive planning, optimization, and management of upcoming fiber-optic networks.

## References

[1] Lau, A. P. T. & Khan, F. N. *Machine Learning for Future Fiber-Optic Communication Systems* (Academic Press, Cambridge, USA, 2022).

[2] Khan FN, Fan Q, Lu C, Lau APT (2019). An optical communication’s perspective on machine learning and its applications. IEEE/OSA J. Light. Technol..

[3] Genty G (2020). Machine learning and applications in ultrafast photonics. Nat. Photonics.

[4] Malkiel, I. et al. Plasmonic nanostructure design and characterization via deep learning. *Light Sci. Appl*. **7**, 60, (2018). 10.1038/s41377-018-0060-7 PMC6123479 30863544

[5] Ma W (2020). Deep learning for the design of photonic structures. Nat. Photonics.

[6] Kojima, K., Koike-Akino, T., Tang, Y., & Wang, Y. Inverse design for integrated photonics using deep neural network. In *Integrated Nanophotonics: Platforms, Devices, and Applications* (eds Yu, P., Xu, H., & Wang, Z.), Ch. 6, 209−243 (Wiley-VCH, Weinheim, Germany, 2023).

[7] Koike-Akino, T. et al. Bayesian optimization for nested adversarial variational autoencoder in tunable nanophotonic device design. In *Proc. Conf. Lasers Electro Opt*., Paper FW4C.7 (San Jose, CA, USA, 2023).

[8] Zhang S (2019). Field and lab experimental demonstration of nonlinear impairment compensation using neural networks. Nat. Commun..

[9] Fan Q, Zhou G, Gui T, Lu C, Lau APT (2020). Advancing theoretical understanding and practical performance of signal processing for nonlinear optical communications through machine learning. Nat. Commun..

[10] Luo S, Soman SKO, Lampe L, Mitra J (2023). Deep learning-aided perturbation model-based fiber nonlinearity compensation. IEEE/OSA J. Light. Technol..

[11] Valkanis A, Papadimitriou G, Beletsioti G, Varvarigos E, Nicopolitidis P (2022). Efficiency and fairness improvement for elastic optical networks using reinforcement learning-based traffic prediction. J. Opt. Commun. Netw..

[12] Qin, X., Hu, Q., Huo, X. & Xie, J. Traffic prediction based on P-ConvLSTM in optical transport networks. In *Proc. Opt. Fiber Commun*., Paper W4G.7 (San Diego, CA, USA, 2023).

[13] Gordon, J. et al. Summary: Workshop on Machine Learning for Optical Communication Systems. NIST Special Publication 2100-04. 10.6028/NIST.SP.2100-04 (2020).

[14] McElligott, T. *Autonomous Networks: Business and Operational Drivers*. TM Forum Research Report. https://inform.tmforum.org/research-and-analysis/reports/autonomous-networks-business-and-operational-drivers (2021).

[15] Ayoub O (2023). Towards explainable artificial intelligence in optical networks: the use case of lightpath QoT estimation. J. Opt. Commun. Net..

[16] Pointurier Y (2021). Machine learning techniques for quality of transmission estimation in optical networks. J. Opt. Commun. Netw..

[17] Yousefi S (2023). Forecasting lightpath quality of transmission and implementing uncertainty in the forecast models. IEEE/OSA J. Light. Technol..

[18] Poggiolini P (2014). The GN-model of fiber non-linear propagation and its applications. IEEE/OSA J. Light. Technol..

[19] Carena A (2014). EGN model of non-linear fiber propagation. Opt. Express.

[20] Lu J (2021). Performance comparisons between machine learning and analytical models for quality of transmission estimation in wavelength-division-multiplexed systems. J. Opt. Commun. Net..

[21] Karandin O, Ferrari A, Musumeci F, Pointurier Y, Tornatore M (2023). Probabilistic low-margin optical-network design with multiple physical-layer parameter uncertainties. J. Opt. Commun. Net..

[22] Khan FN (2023). Data perspectives in AI-assisted fiber-optic communication networks. IEEE Netw..

[23] Perrin, S. *Open, Automated, & Programmable Transport Networks: A 2022 Heavy Reading Survey.* Heavy Reading White Paper. https://www.infinera.com/white-paper/open-automated-programmable/ (2022).

[24] Dong Z (2016). Optical performance monitoring: a review of current and future technologies. IEEE/OSA J. Light. Technol..

[25] Saif WS (2020). Machine learning techniques for optical performance monitoring and modulation format identification: a survey. IEEE Commun. Surv. Tut..

[26] Tanimura T, Hoshida T, Kato T, Watanabe S, Morikawa H (2019). Convolutional neural network-based optical performance monitoring for optical transport networks. J. Opt. Commun. Netw..

[27] Cooperson, D. *New-generation Intelligent Operations: The Service-centric Transformation Path.* TM Forum Research Report. https://inform.tmforum.org/research-and-analysis/reports/new-generation-intelligent-operations-the-service-centric-transformation-path (2023).

[28] Morris, I. Colt’s AI trials provide good news for humans. https://www.lightreading.com/artificial-intelligence-machine-learning/colts-ai-trials-provide-good-news-for-humans/d/d-id/750748.

[29] Jovanovic, O., Ros, F. D., Yankov, M., & Zibar, D. End-to-end learning for fiber-optic communication systems. In *Machine Learning for Future Fiber-Optic Communication Systems* (eds Lau, A. P. T. & Khan, F. N.), Ch. 5, 115–139 (Academic Press, Cambridge, USA, 2022).

[30] Karanov B (2018). End-to-end deep learning of optical fiber communications. IEEE/OSA J. Light. Technol..

[31] Niu Z (2022). End-to-end deep learning for long-haul fiber transmission using differentiable surrogate channel. IEEE/OSA J. Light. Technol..

[32] Microsoft data set, Wide-area optical backbone performance. https://www.microsoft.com/en-us/research/project/microsofts-wide-area-optical-backbone/.

[33] Zhai, Z. Alibaba-cloud-transport-system data set, GitHub. https://github.com/alibaba/alibaba-cloud-transport-system (2023).

[34] http://sndlib.zib.de/home.action Germany50 and GÉANT data sets, Networks with multiple demand matrices.

[35] Furdek M (2020). Machine learning for optical network security monitoring: a practical perspective. IEEE/OSA J. Light. Technol..

[36] Natalino C, Schiano M, Giglio AD, Furdek M (2022). Root cause analysis for autonomous optical network security management. IEEE Trans. Netw. Serv. Manag..

[37] Furdek M, Natalino C, Giglio AD, Schiano M (2021). Optical network security management: requirements, architecture, and efficient machine learning models for detection of evolving threats,. J. Opt. Commun. Netw..

[38] Lemaire, V. et al. Proactive fiber break detection based on quaternion time series and automatic variable selection from relational data. In *Proc. Int. Workshop on Advanced Analysis and Learning on Temporal Data (AALTD)*, 26–42 (Würzburg, Germany, 2019).

[39] Mackert, M., Scholz, M. & Mikoleit, M. AI engineering and usage – Deutsche Telekom professional ethics. https://www.telekom.com/en/company/digital-responsibility/details/what-that-means-for-our-employees-637074.

[40] Wang D (2022). A review of machine learning-based failure management in optical networks. Sci. China Inf. Sci..

[41] Lun H (2023). A GAN based soft failure detection and identification framework for long-haul coherent optical communication systems. IEEE/OSA J. Light. Technol..

[42] Boitier, F. et al. Proactive fiber damage detection in real-time coherent receiver. In *Proc. Eur. Conf. Opt. Commun*., Gothenburg, Sweden, Paper Th.2.F.1 (2017).

[43] Unified architecture for machine learning in 5G and future networks, Technical Specification ITU-T FG-ML5G-ARC5G. https://www.itu.int/en/ITU-T/focusgroups/ml5g/Documents/ML5G-delievrables.pdf.

[44] Luo, S. Improved operator experience through experiential networked Intelligence (ENI), ETSI White Paper No. 22. https://www.etsi.org/images/files/ETSIWhitePapers/etsi_wp22_ENI_FINAL.pdf.

[45] Ayassi R, Triki A, Crespi N, Minerva R, Laye M (2022). Survey on the use of machine learning for quality of transmission estimation in optical transport networks. IEEE/OSA J. Light. Technol..

[46] Whitesmith M (2020). Cognitive Bias in Intelligence Analysis.

[47] BEREC report on the impact of artificial intelligence (AI) solutions in the telecommunications sector on regulation, BEREC Report BoR (23) 93. https://www.berec.europa.eu/en/document-categories/berec/reports.

[48] Amar, J., Lajous, T., Majumder, S. & Surak, Z. How AI is helping revolutionize telco service operations. https://www.mckinsey.com/industries/technology-media-and-telecommunications/our-insights/how-ai-is-helping-revolutionize-telco-service-operations.

[49] Tanimura T, Yoshida S, Tajima K, Oda S, Hoshida T (2021). Concept and implementation study of advanced DSP-based fiber-longitudinal optical power profile monitoring toward optical network tomography. J. Opt. Commun. Netw..

[50] Khan FN (2017). Joint OSNR monitoring and modulation format identification in digital coherent receivers using deep neural networks. Opt. Express.

[51] VPIphotonics, VPItransmissionMaker^TM^ Optical Systems. https://www.vpiphotonics.com/Tools/OpticalSystems/.

[52] Yang, Q., Zhang,Y., Dai, W. & Pan, S. J. *Transfer Learning* (Cambridge University Press, Cambridge, UK, 2020).

[53] European Union-funded MENTOR project, “Machine learning in optical networks,” https://cordis.europa.eu/project/id/956713.

[54] Nakayama, K. & Jeno, G. *Federated Learning with Python* (Packt Publishing, Birmingham, UK, 2022).

[55] Simeone O (2022). Machine Learning for Engineers.

[56] Samek W, Montavon G, Lapuschkin S, Anders CJ, Müller K-R (2021). Explaining deep neural networks and beyond: a review of methods and applications. Proc. IEEE.

[57] Kim B, Doshi-Velez F (2021). Machine learning techniques for accountability. AI Mag..

[58] The use of supplier’s declaration of conformity, NIST document https://www.nist.gov/system/files/documents/standardsgov/Sdoc.pdf.

[59] Calmon, F., Wei, D., Vinzamuri, B., Ramamurthy, K. N. & Varshney, K. R. Optimized pre-processing for discrimination prevention. In *Proc. Neural Information Processing Systems*, 3995–4004 (Long Beach, CA, USA, 2017).

[60] Kamishima, T., Akaho, S. H. Asoh, and J. Sakuma, Fairness-aware classifier with prejudice remover regularizer. In *Proc. Joint European Conference on Machine Learning and Knowledge Discovery in Databases*, 35–50 (Bristol, UK, 2012).

[61] Hardt, M., Price, E. & Srebro, N. Equality of opportunity in supervised learning. In *Proc. Neural Information Processing Systems*, 3315–3323 (Barcelona, Spain, 2016).

[62] Yu J, Zhu S, Gutterman CL, Zussman G, Kilper DC (2021). Machine-learning-based EDFA gain estimation. J. Opt. Commun. Netw..

[63] de Moura UC, Ros FD, Brusin AMR, Carena A, Zibar D (2021). Experimental characterization of Raman amplifier optimization through inverse system design. IEEE/OSA J. Light. Technol..

[64] Fernandez, S. H., Jovanovic, O., Peucheret, C., Ros, F. D. & Zibar, D. Differentiable machine learning-based modeling for directly-modulated lasers. *IEEE Photon. Technol. Lett*. **36**, 266–269 (2024).

[65] Zibar D (2019). Highly-sensitive phase and frequency noise measurement technique using Bayesian filtering. IEEE Photon. Technol. Lett..

[66] Wiecha PR, Arbouet A, Girard C, Muskens OL (2021). Deep learning in nano-photonics: inverse design and beyond. Photon. Res..

[67] Sanchez M, Everly C, Postigo PA (2024). Advances in machine learning optimization for classical and quantum photonics. J. Opt. Soc. Am. B.

[68] Jiang M (2022). Fiber laser development enabled by machine learning: review and prospect. PhotoniX.

[69] Newman, M. & Ramsay, D. Mapping a path to telco revenue growth, Oct. 2021, TM Forum Research Report. https://inform.tmforum.org/research-and-analysis/reports/mapping-a-path-to-telco-revenue-growth.

[70] Li X-H (2022). A survey of data-driven and knowledge-aware explainable AI. IEEE Trans. Knowl. Data Eng..

[71] Yang, Y., Liu, Y. & Naghizadeh, P. Adaptive data debiasing through bounded exploration. In *Proc. Neural Information Processing Systems*, 1516–1528 (New Orleans, LA, USA, 2022).

[72] Zhang, B. H., Lemoine, B. & Mitchell, M. Mitigating unwanted biases with adversarial learning. In *Proc. AAAI/ACM Conf. AI, Ethics, and Society*, 335–340 (New Orleans, LA, USA, 2018).

[73] Panayiotou T, Michalopoulou M, Ellinas G (2023). Survey on machine learning for traffic-driven service provisioning in optical networks. IEEE Commun. Surv. Tut..

[74] Nevin JW (2022). Techniques for applying reinforcement learning to routing and wavelength assignment problems in optical fiber communication networks. J. Opt. Commun. Netw..

[75] Di Cicco, N. et al. On deep reinforcement learning for static routing and wavelength assignment. *IEEE J. Sel. Top. Quantum Electron*. **28**, 3600112 (2022).

[76] Xiang J (2023). Low-complexity conditional generative adversarial network (c-GAN) based nonlinear equalizer for coherent data-center interconnections. IEEE/OSA J. Light. Technol..

[77] Bluemm C (2023). Hardware-efficient duobinary neural network equalizers for 800 Gb/s IM/DD PAM4 transmission over 10 km SSMF. IEEE/OSA J. Light. Technol..

